# Effective connectivity between deep brain stimulation targets in individuals with treatment-resistant depression

**DOI:** 10.1093/braincomms/fcad256

**Published:** 2023-10-07

**Authors:** Saba Amiri, Mohammad Arbabi, Milad Rahimi, Mansour Parvaresh-Rizi, Mehdi M Mirbagheri

**Affiliations:** Neuroscience Research Center, Shahid Beheshti University of Medical Science, Tehran 1983969367, Iran; Psychiatry, Psychosomatic Medicine Research Center Department, Tehran University of Medical Sciences, Tehran 1419733141, Iran; Medical Physics and Biomedical Engineering Group, Faculty of Medicine, Tehran University of Medical Sciences (TUMS), Tehran 1461884513, Iran; Neurosurgery Department, Iran University of Medical Sciences (IUMS), Tehran 02166509120, Iran; Medical Physics and Biomedical Engineering Group, Faculty of Medicine, Tehran University of Medical Sciences (TUMS), Tehran 1461884513, Iran; Physical Medicine and Rehabilitation Department, Northwestern University, Chicago IL 60611, USA; Neural Engineering and Rehabilitation Research Center, Tehran 1146733711, Iran

**Keywords:** effective connectivity, deep brain stimulation, depression, treatment

## Abstract

The therapeutic effect of deep brain stimulation on patients with treatment-resistant depression is strongly dependent on the connectivity of the stimulation region with other regions associated with depression. The aims of this study are to characterize the effective connectivity between the brain regions playing important roles in depression and further investigate the underlying pathophysiological mechanisms of treatment-resistant depression and the mechanisms involving deep brain stimulation. Thirty-three individuals with treatment-resistant depression and 29 healthy control subjects were examined. All subjects underwent resting-state functional MRI scanning. The coupling parameters reflecting the causal interactions among deep brain stimulation targets and medial prefrontal cortex were estimated using spectral dynamic causal modelling. Our results showed that compared to the healthy control subjects, in the left hemisphere of treatment-resistant depression patients, the nucleus accumbens was inhibited by the inferior thalamic peduncle and excited the ventral caudate and the subcallosal cingulate gyrus, which in turn excited the lateral habenula. In the right hemisphere, the lateral habenula inhibited the ventral caudate and the nucleus accumbens, both of which inhibited the inferior thalamic peduncle, which in turn inhibited the cingulate gyrus. The ventral caudate excited the lateral habenula and the cingulate gyrus, which excited the medial prefrontal cortex. Furthermore, these effective connectivity links varied between males and females, and the left and right hemispheres. Our findings suggest that intrinsic excitatory/inhibitory connections between deep brain stimulation targets are impaired in treatment-resistant depression patients, and that these connections are sex dependent and hemispherically lateralized. This knowledge can help to better understand the underlying mechanisms of treatment-resistant depression, and along with tractography, structural imaging, and other relevant clinical information, may assist to determine the appropriate region for deep brain stimulation therapy in each treatment-resistant depression patient.

## Teaser

Amiri *et al*. identified abnormal effective connectivity between deep brain stimulation targets in treatment-resistant depression patients with impaired excitatory and inhibitory connections. Effective connectivity patterns varied across hemispheres and genders, emphasizing the need for individualized deep brain stimulation targeting in effective treatment-resistant depression therapy.

## Graphical Abstract

**Graphical Abstract:**
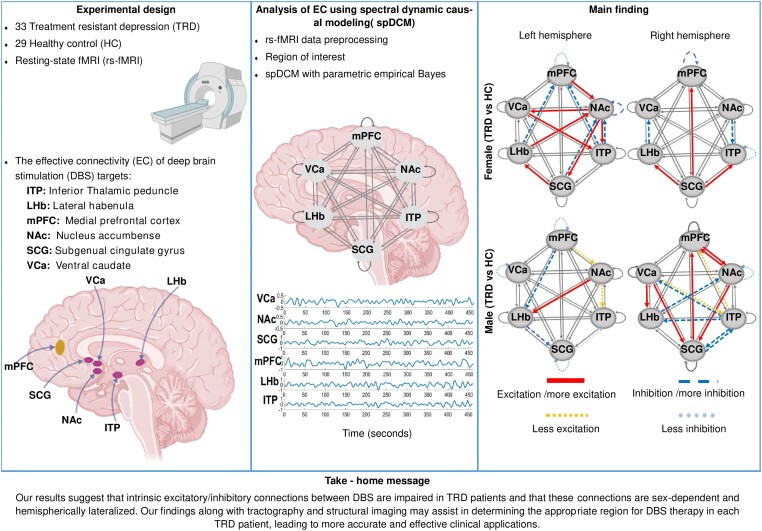

## Introduction

Major depression disorder (MDD) is one of the most common psychiatric diseases with a lifetime prevalence of ∼4.7% in the general population, ranging from 2 to 21% with the highest rates found in some European countries and the lowest in some Asian countries.^1,2^ An accurate diagnosis, effective treatment and prevention of the disorder have yet to be addressed due to its complexity and inadequate knowledge of the primary underlying mechanisms. Moreover, around one-third of MDD patients do not react to common treatments including antidepressant medications and psychological interventions, known as treatment-resistant depression (TRD) patients.^3–6^ In later decades, deep brain incitement (DBS) has been created and utilized to treat TRD with a ∼60% responder rate.^7–9^ For TRD treatment, the major challenge of using DBS is determining the most appropriate brain region for electrode implantation, which requires the characterization of structural and functional brain networks associated with DBS.

Recent studies show that functional neuroimaging may help address key questions about the aetiology and pathophysiology of depression. Neuroimaging studies based on blood-oxygen-level-dependent functional MRI (fMRI) have revealed that depression can be characterized as a disorder with disrupted functional connectivity among various brain regions and networks including the default mode network,^10–12^ executive control network and salience network.^13,14^ For example, Accolla *et al*.^15^ found stronger connectivity of subcallosal cingulate gyrus (SCG; brain target DBS) to the medial prefrontal cortex (mPFC) in TRD subjects treated with SCG-DBS compared to those who were non-responders, whereas altered functional connectivity to mPFC was reported in these patients.^16–18^ Although functional connectivity sheds light on this issue, it is incapable of providing causal relationships between brain functional connectivity abnormalities and depression. This deficit can be compensated by using effective connectivity (EC), which can measure the causal relationship between target regions and characterize their inhibitory/excitatory impact on each other.

To measure EC, dynamic causal modelling (DCM) has been largely used.^19^ However, few studies of DCM applications have been performed mostly on limited regions in patients with major depression under resting-state fMRI.^20–23^ A decreased EC from the left parietal cortex to other default mode network regions,^20^ from the anterior insula to the middle frontal gyrus, and an increased EC from the amygdala to the anterior insula have been reported.^21^ A recent study on a larger-scale network, including default mode, executive control, salience and limbic networks, has observed the coexistence of the reduced excitatory and increased inhibitory causal connections in the default mode network.^23^

DCM was found to more accurately model the neural coupling of fMRI data among different EC methods including the Granger causality analysis,^24^ structure equation modelling^25^ and psychophysiological interaction.^26^ The application of DCM was limited to the analysis of task-related fMRI data but recently extended to resting-state fMRI as well. The DCM model for resting-state fMRI can be evaluated utilizing stochastic or spectral DCM.^26^ The spectral DCM estimates EC based on correlation functions in the frequency domain, which is more computationally efficient and sensitive to group differences (e.g. patients and controls), as compared to stochastic DCM. In this study, we aimed to characterize the abnormality in EC between DBS targets in TRD patients using spectral DCM. The findings help better understand the mechanisms underlying depression in TRD patients.

There are numerous evidences indicating sex differences among certain biological variables in depressive patients. Males and females with MDD differ in monoaminergic, neuroplasticity and immune system markers as well as in some hormones and neurotransmitters.^27,28^ Several studies focusing on the functional connectivity of male and female subjects together obviate the possibility of finding sex-specific FC changes in patients with depression. However, recent studies of brain connectivity have shown sex differences in cognitive patterns,^29,30^ emotion regulation^31^ and neural stress responses.^32^ Therefore, sex differences play an important role in this regard and require further evaluation to provide for better understanding the mechanisms underlying EC abnormalities in the TRD group. To investigate the influence of sex dependence on the TRD and healthy control (HC) groups, we studied the alterations in ECs between the male and female TRD groups.

## Materials and methods

### Subjects and clinical assessments

Thirty-three people with TRD and 29 healthy control subjects were examined. The TRD group (12M) and the control group (13M) had a mean ± SD age of 34.6 ± 8.8 years and 31.4 ± 6.3 years, respectively. The TRD group had a disease duration of 10 ± 3.8 years (mean ± SD). Treatment resistance was defined as the inability to exhibit a satisfactory response to at least two antidepressant courses with standard dosage and duration (at least 6 weeks for each treatment course).^33^ The patients were asked to be antidepressant free for at least 2 weeks before the study. The severity of the current episode was assessed using the 17-item Hamilton Depressive Rating Scale,^34^ and a total score of at least 20 was the cut-off for inclusion. To avoid inter-examiner inconstancy, the same therapist met and assessed all subjects. Subjects were excluded if they had a second diagnosis (psychotic, anxiety and substance-related disorder), severe decompensated somatic disorder, neurological disorder and history of head trauma with loss of consciousness. It is important to note that despite its widespread clinical use, the precise mechanisms underlying the therapeutic effects of electroconvulsive therapy (ECT) are not fully understood.^35,36^ ECT has a global impact on the entire brain rather than targeting specific regions or circuits. Consequently, it can induce extensive changes in both global and local brain connectivity and activity.^37,38^ These changes can complicate and disrupt our understanding of the mechanisms involved in TRD patients. To ensure the reliability of our study results regarding EC and the mechanisms involved in TRD patients, we specifically excluded individuals with a history of ECT therapy as one of the criteria for participation. This exclusion allowed us to focus on the specific factors related to EC in TRD patients without the confounding effects of prior ECT treatment. All subjects gave educated consent to the experimental procedure that was reviewed and approved by Tehran University’s Research Ethics Board.

### Image acquisition

Multimodal MRI data were collected using a Siemens Magnetom Prisma 3T MRI scanner. The resting-state fMRI images covering the whole brain were obtained with an echo-planar imaging sequence with the following parameters: volume = 240, axial slices = 32, slices thickness = 3.5 mm, repetition time = 2000 ms, echo time = 30 ms, flip angle = 90°, voxel size = 3.1 × 3.1 × 3.5 mm, field of view = 200 × 200 mm, and matrix size = 64 × 64. T_1_-weighted structural images were acquired for co-registration of functional images using a sagittal 3D magnetization-prepared rapid acquisition gradient echo sequence: repetition time = 1800 ms, echo time = 3.53 ms, inversion time = 1100 ms, flip angle = 7°, field of view (FOV) = 256 × 256 mm^2^, matrix size **=** 256 × 256, slice thickness = 1 mm, scan time (magnetization-prepared rapid acquisition gradient echo) = 4 min and 12 s, and scan time (resting-state fMRI) = 8 min and 6 s. All subjects were awake and asked to keep their eyes closed during resting-state functional imaging.

### fMRI processing and statistical analysis

Using a combination of the data processing assistant for resting-state fMRI (DPARSF) toolbox version 4.5^39^ and statistical parametric mapping (SPM12)^40^ software, we conducted data analyses including preprocessing resting-state fMRI data, defining regions of interest (ROIs), extracting ROI time series using a general linear model, defining and estimating DCM to examine EC and carrying out between-group analyses using parametric empirical Bayes (PEB). PEB is a novel approach that permits group comparisons across model parameters utilizing both posterior expectations and posterior uncertainties of the model.^41^

### Preprocessing

We performed the resting-state fMRI data preprocessing using SPM12 and the DPARSF toolbox version 4.5.^39^ Briefly, the following steps were carried out:

- Removing the first 10 volumes of the 240 volumes to allow for magnetization equilibrium.
- Skull stripping of both functional and structural images to remove non-brain tissue before co-registration of T_1_ images and functional images for better registration of T_1_ image to functional space. For skull stripping, we used the BET toolbox in DPARSF and checked manually.
- Slice timing correction.
- Correcting for head movements and any subject who had a maximum displacement > 2 mm, a maximum rotation >2.0° or a mean framewise displacement > 0.2 was excluded from the group. By checking the eligibility criteria, one of the subjects was excluded from the analyses, which required the images to be realigned with a six-parameter (rigid body) linear transformation. Individual structural images were co-registered to mean functional images.
- Performing segmentation of T_1_-weighted images to grey matter, white matter and CSF.
- Regressing out 29 nuisance covariates, including 5 eigenvariates from white matter, CSF and Friston 24 motion parameters.
- Converting spatial normalization into the standard template, Montreal Neurological Institute (MNI) space.
- Smoothing using a Gaussian filter with full width at half maximum (FWHM) = 4 mm. We have chosen this smoothing kernel based on recent similar studies.^42^
- Temporal band-pass filtering (0.01–0.01 Hz) to reduce the influence of low-frequency drift and high-frequency physiological noise. The band-pass filtering and nuisance removal were completed simultaneously in the analysis, which avoided a potential artefact re-introduced by modular preprocessing.

### ROI definition

For each subject, six ROIs were chosen in both hemispheres based on the Brainnetome atlas,^43^ consisting of five DBS targets: the SCG,^7,44^ ventral caudate (VCa),^45^ nucleus accumbens (NAc),^46^ inferior thalamic peduncle (ITP),^47^ lateral habenula (LHb)^48,49^ and one potentially associated with these targets: the mPFC. The detailed MNI coordinates of these ROIs are provided in Table 1.

**Table 1:** ROIs and MNI coordinates in the DCM

Regions of interest name	MNI coordinate Left hemisphere	MNI coordinate Right hemisphere
Subcallosal cingulate gyrus (SCG)	−4, 39, −2	5, 41, 6
Ventral caudate (VCa)	−12, 14, 0	15, 14, −2
Nucleus accumbens (NAc)	−17, 3, −9	15, 8, −9
Inferior thalamic peduncle (ITP)	−7, −14, 7	3, −13, 5
Lateral habenula (LHb)	−7, −12, 5	7, −11, 6
Medial prefrontal cortex (mPFC)	−7, 54, −7	6, 47, −7

DCM is an analysis technique that aims to infer the causal architecture of coupled or distributed dynamical systems. This is performed by using a Bayesian model that compares models of how time series data were generated. Firstly, DCM builds a random model of causality between different parts of the system. Secondly, it gradually updates this model by using the Bayesian role with respect to each data. Finally, DCM will have an estimate of the causal architecture of the system. In the fMRI field, a model with maximum similarity of its time series to the blood-oxygen-level-dependent (BOLD) signal of the brain regions is the best fitted model and can be interpreted as having EC between brain regions. EC, in contrast to functional connectivity, is a directed graph that shows the causal relationship between nodes, which in turn demonstrates whether each edge is inhibitory or excitatory. In other words, DCM, using BOLD signals, estimates a directional model of the interaction between brain regions. This model can explain how the output of a specific region of the brain affects another region. If the particular region inhibits another region, its connection weight to that region is negative, and if it excites it, the weight is positive (see Friston *et al.*^50^ for more details).

### Spectral DCM with PEB

**Figure 1:**
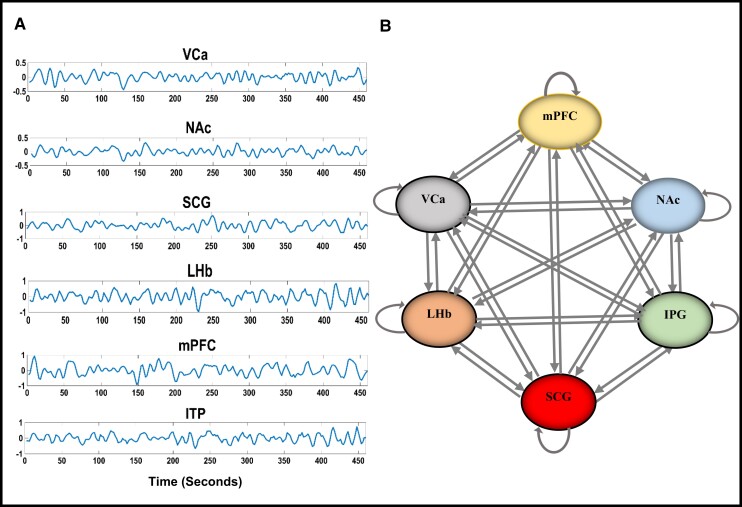
**Fully connected model.** (**A**) Blood-oxygen-level-dependent (BOLD) time series data from the ROIs of one subject. (**B**) A schematic model showing the fully connected model with six regions. The fully connected model including bi-directional connections between all pairs of ROIs and self-inhibitory connections for each region. SCG, subcallosal cingulate gyrus; VCa, ventral caudate; NAc, nucleus accumbens; ITP, inferior thalamic peduncle; LHb, lateral habenula; mPFC, medial prefrontal cortex.

The spectral DCM (spDCM) within the framework of the PEB analysis was performed by DCM12 implemented in SPM12, for each hemisphere, separately. First, after selecting six ROI regions, we performed a generalized linear model analysis to extract ROI-specific time series from the original preprocessed data for DCM analysis. We assumed that all participants used the same model and specified a fully connected model consisting of six predefined ROIs in each brain hemisphere for each subject. Fully connected models mean that each ROI was assumed to be connected to all other regions (Fig. 1). Second, parameters of the fully connected model were then estimated for each participant with a PEB that alternated the estimation of DCMs at the first level (within-participant) by estimating the group effect. Third, after estimating all parameters of the fully connected models using Bayesian model reduction, any connectivity parameter from the group-level PEB that did not contribute to model evidence was pruned away. Fourth, the Bayesian model averaging of the best-pruned model parameters is applied at group level prior to averaging the subject-specific DCMs, and the stability of the individually estimated DCMs was checked by computing the Lyapunov exponent of each endogenous connectivity matrix, which provides a mathematical degree of stability of a differential equation system’s solution^51,52^; therefore, the winning model is empirically determined.

### Statistical analyses

We thought about the connectivity parameters of within-group and between-group effects with a posterior probability (PP) > 0.99 (*P* > 0.99) to be significant. One approach to identify the differences between the two groups is conducting a classical test on DCM connectivity parameters (e.g., *t*-tests). The disadvantage of this approach is that it ignores the estimated uncertainty (variance) about the connection strength. Instead, a Bayesian hierarchical model may be made for the parameters, describing however group-level effects constrain parameter estimates on a subject basis. Moreover, we used Bayesian statistics in which the statistical probability of one model is compared and tested with other models without using *P*-values. Correction for multiple comparisons is not required in DCM as there are no null hypotheses normally tested. Rather, the probability estimating the connectivity parameter, lying within a certain range of value, is computed.^19^ Finally, to assess the potential impact of duration and severity of treatment resistance on EC measures, we performed Pearson’s correlation coefficients using SPSS 25(SPSS Inc., Chicago, IL).

## Results

**Figure 2:**
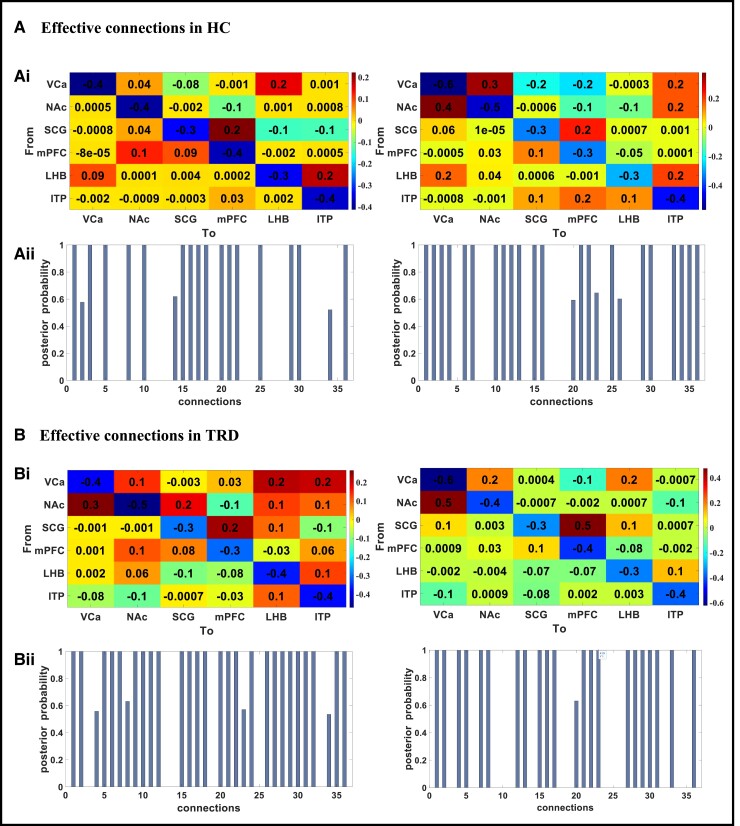
**Average group EC in the HC and TRD groups.** (**Ai**) The EC matrix in the HC group. (**Aii**) PP of 36 connections corresponding to the EC matrix for the HC group. (**Bi**) The EC matrix in the TRD group. (**Bii**) PP of 36 connections corresponding to the EC matrix for the TRD group. Thresholding for inference with a PP of 99% was used. VCa, ventral caudate; NAc, nucleus accumbens; ITP, inferior thalamic peduncle; LHb, lateral habenula; mPFC, medial prefrontal cortex; EC, effective connectivity; HC, healthy control; TRD, treatment-resistant depression; PP, posterior probability.

**Figure 3:**
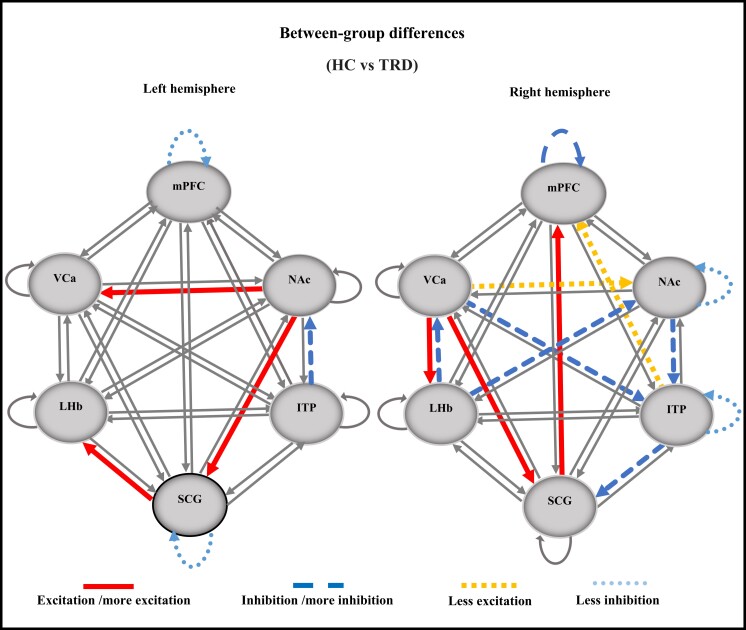
**ECs with significant differences between the TRD and HC groups.** The left hemisphere and the right hemisphere. Comparing the TRD group to the HC group: non-significant connections (thin continuous lines), excitation/more excitatory connections in TRD compared to HC (thick continuous lines), less excitatory connections in TRD compared to HC (square dotted lines), inhibition/more inhibitory connections in TRD compared to HC (dashed lines) and less inhibitory connections in TRD compared to HC (dotted lines). VCa, ventral caudate; NAc, nucleus accumbens; ITP, inferior thalamic peduncle; LHb, lateral habenula; mPFC, medial prefrontal cortex; HC, healthy control; TRD, treatment-resistant depression.

EC was individually estimated for all participants using the spectral DCM approach. From the fully connected model, ECs between DBS targets and mPFC were identified in both hemispheres of each individual in the TRD and HC groups. Figure 2 shows the average group results of spDCM for these regions. A PP of 99% was used as a threshold for reasoning. The connections with a PP of >99% were thought about as significant edges and shown with a bigger font for the HC (Fig. 2Ai) and TRD (Fig. 2Bi) groups. The results indicated many significant alterations within the ECs of each hemisphere for the TRD groups that were additionally investigated in Table 2 and Fig. 3.

**Table 2:** Average effective connectivity with significant edges in TRD and HC groups

Left connections	TRD	HC	Right connections	TRD	HC
L-NAc → L-VCa	0.3	0.0005	R-VCa → R-NAc	0.2	0.3
L-NAc → L-SCG	0.2	−0.002	R-VCa → R-SCG	0.0004	−0.2
L-SGC → L-SCG	−0.2	−0.3	R-VCa → R-LHb	0.2	−0.0003
L-SCG → L-LHb	0.1	−0.1	R-VCa → R-ITP	−0.0007	0.2
L-mPFC → L-mPFC	−0.3	−0.4	R-NAc → R-NAc	−0.4	−0.5
L-ITP → L-NAc	−0.1	−0.0009	R-NAc → R-ITP	−0.1	0.2
			R-SCG → R-mPFC	0.5	0.2
			R-mPFC → R-mPFC	−0.4	−0.3
			R-LHb → R-VCa	−0.0002	0.2
			R-LHb → R-NAc	−0.004	0.04
			R-ITP → R-SCG	−0.08	0.1
			R-ITP → R-mPFC	0.002	0.2

### EC between selected brain regions in the TRD and HC groups

#### The left hemisphere

Table 2 summarizes the EC analysis results, showing ECs with significant differences that vary between the HC and TRD groups. In the left hemisphere, connections from NAc to SCG and SCG to LHb were excitatory in the TRD group, whereas they were inhibitory in the HC group. By comparing the two groups, several ECs with significant differences in the left hemisphere were identified, as shown in Fig. 3 and described below. In patients with TRD, compared to the HC group, the following major EC links were detected: (i) ITP inhibited NAc; (ii) NAc excited both SCG and VCa; and (iii) SCG excited LHb.

#### The right hemisphere

ECs with significant differences, which were altered between the HC and TRD groups, are shown in Table 2. In the right hemisphere, EC links including LHb to NAc and VCa, VCa to ITP and NAc to ITP were inhibitory intrinsic connectivity in the TRD group and excitatory intrinsic connectivity in the HC group. On the other hand, the ECs between VCa to SCG and LHb were excitatory intrinsic connectivity in the TRD group and inhibitory intrinsic connectivity in the HC group.

Figure 3 shows ECs with significant differences in the right hemisphere between the TRD and HC groups. Compared to the HC group, in the TRD group, the following points arose: (i) LHb inhibited VCa and NAc, both of which inhibited ITP; (ii) ITP inhibited SCG, which excited mPFC; and (iii) VCa excited both SCG and LHb.

### Sex dependence and hemispheric lateralization

The nature of abnormalities in EC may be diverse between the male and female groups. Therefore, our results could be sex biased since the number of TRD females in our study was more than twice the number of TRD males, reliable with the TRD population. To investigate the influence of sex dependence and hemispheric lateralization on the TRD and HC groups, we studied the alterations in ECs between the male and female TRD groups.

### Female groups

**Figure 4:**
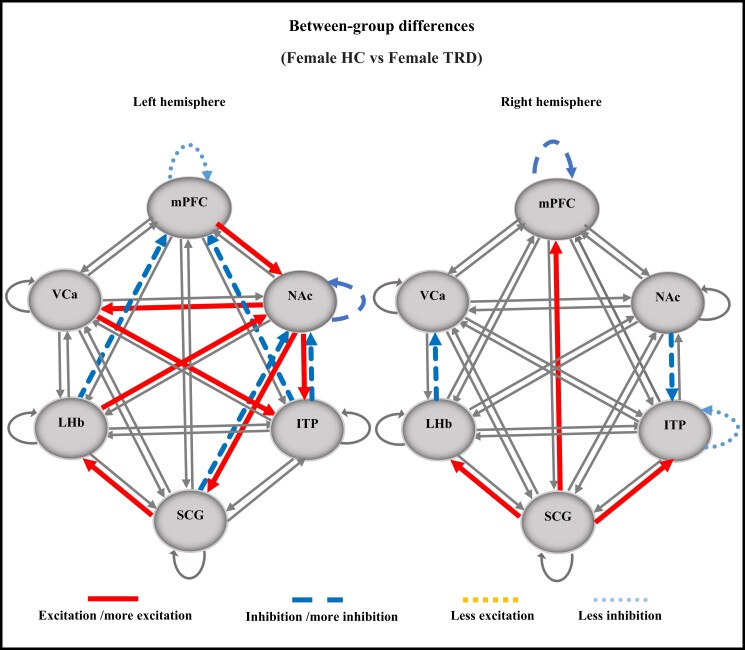
**ECs with significant differences between the female TRD and HC groups.** The left hemisphere and the right hemisphere. Comparing the TRD group to the HC group: non-significant connections (thin continuous lines), excitation/more excitatory connections in TRD compared to HC (thick continuous lines), less excitatory connections in TRD compared to HC (square dotted lines), inhibition/more inhibitory connections in TRD compared to HC (dashed lines) and less inhibitory connections in TRD compared to HC (dotted lines). VCa, ventral caudate; NAc, nucleus accumbens; ITP, inferior thalamic peduncle; LHb, lateral habenula; mPFC, medial prefrontal cortex; HC, healthy control; TRD, treatment-resistant depression.

Table 3 shows the average group ECs with significant edges in females in both the TRD and HC groups. In the left hemisphere of patients with TRD, the EC links included NAc to SCG, VCa and ITP; LHb to NAc; SCG to LHb; and VCa to ITP. The inhibitory intrinsic links included ITP to NAc; ITP to mPFC; SCG to NAc; and LHb to mPFC. Interestingly, all inhibitory intrinsic ECs in the TRD group were excitatory intrinsic ECs in the HC group and vice versa with the exception of EC from mPFC to NAc, which was excitatory in both groups. Figure 4 shows ECs with significant differences in the left hemisphere between the female TRD and HC groups. In the TRD group, compared to the HC group, the following points emerged: (i) NAc was inhibited by SCG and ITP but excited by mPFC and LHb; (ii) NAc excited VCa, ITP and SCG, which in turn excited LHb; and (iii) mPFC was inhibited by both ITP and LHb.

**Table 3:** Average effective connectivity with significant edges in female TRD and HC groups

Left connections	TRD	HC	Right connections	TRD	HC
L-VCa → L-ITP	0.3	−0.0002	R-NAc → R-ITP	−0.2	0.001
L-NAc → L-VCa	0.3	−0.001	R-SCG → R-mPFC	0.4	0.1
L-NAc → L-NAc	−0.5	−0.4	R-SCG → R-LHb	0.1	−0.04
L-NAc → L-SGC	0.2	−0.08	R-SCG → R-ITP	0.04	−0.004
L-NAc → L-ITP	0.002	−0.3	R-mPFC → R-mPFC	−0.4	−0.2
L-SGC → L-NAc	−0.004	0.09	R-LHb → R-VCa	−0.2	0.2
L-SGC → L-LHb	0.1	−0.2	R-ITP → R-ITP	−0.3	−0.5
L-LHb → L-NAc	0.08	−0.003			
L-LHb → L-mPFC	−0.2	0.005			
L-mPFC → L-NAc	0.1	0.001			
L-mPFC → L-mPFC	−0.3	−0.4			
L-ITP → L-NAc	−0.1	0.003			
L-ITP → L-mPFC	−0.09	0.001			

In the right hemisphere of the female TRD group, ECs were excitatory intrinsic from SCG to LHb, ITP and mPFC and inhibitory intrinsic from NAc to ITP and LHb to VCa (Table 3). Similar to the left hemisphere, all excitatory intrinsic connections in the right hemisphere of the female TRD group were inhibitory intrinsic ECs in the female HD group and vice versa, except for EC from SCG to mPFC that was excitatory in both female groups. Figure 4 shows ECs with significant differences in the right hemisphere between the two female groups. Interestingly, in the female TRD group as compared to the female HC group, SCG was the only region with excitatory effects: excited LHb, ITP and mPFC. The inhibitory ECs were limited to links from NAc to ITP, LHb to VCa and the self-inhibition of mPFC.

### Male groups

**Figure 5:**
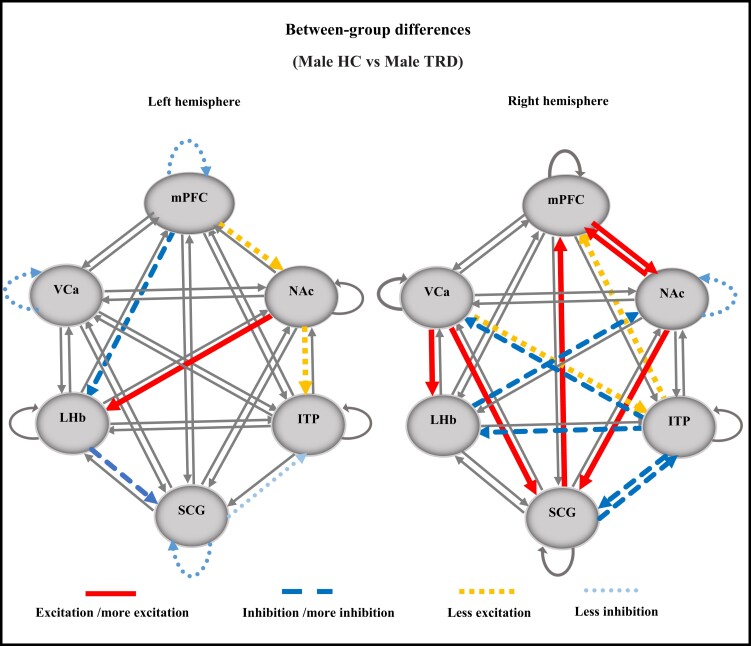
**ECs with significant differences between the male TRD and HC groups.** The left hemisphere and the right hemisphere. Comparing the TRD group to the HC group: non-significant connections (thin continuous lines), excitation/more excitatory connections in TRD compared to HC (thick continuous lines), less excitatory connections in TRD compared to HC (square dotted lines), inhibition/more inhibitory connections in TRD compared to HC (dashed lines) and less inhibitory connections in TRD compared to HC (dotted lines). VCa, ventral caudate; NAc, nucleus accumbens; ITP, inferior thalamic peduncle; LHb, lateral habenula; mPFC, medial prefrontal cortex; HC, healthy control; TRD, treatment-resistant depression.

Table 4 shows the average group ECs with significant edges in males in both the TRD and HC groups. EC links in the left hemisphere of both male groups were excitatory intrinsic from NAc to LHb and ITP and mPFC to NAc and inhibitory intrinsic from SCG to ITP. On the other hand, EC links from mPFC to LHb and LHb to SCG were inhibitory intrinsic in the male TRD group and excitatory intrinsic in the HC group. ECs with significant differences in the right hemisphere between the two female groups are shown in Fig. 5. Compared to the male HC group, the major activity in the male TRD group was related to LHb that was excited by NAc and inhibited by LHb, which in turn inhibited SCG.

**Table 4:** Average effective connectivity with significant edges in male TRD and HC groups

Left connections	TRD	HC	Right connections	TRD	HC
L-VCa → L-VCa	−0.3	−0.4	R-VCa → R-SCG	0.2	−0.1
L-NAc → L-LHb	0.3	0.0002	R-VCa → R-LHb	0.2	−0.06
L-NAc → L-ITP	0.2	0.3	R-VCa → R-ITP	0.06	0.3
L-SGC → L-SGC	−0.25	−0.31	R-NAc → R-mPFC	0.03	−0.2
L-SGC → L-ITP	−0.001	−0.2	R-NAc → R-NAc	−0.4	−0.6
L-mPFC → L-NAc	0.001	0.2	R-NAc → R-SCG	0.0001	−0.06
L-mPFC → L-mPFC	−0.3	−0.4	R-SCG → R-mPFC	0.5	0.3
L-mPFC → L-LHb	−0.1	0.004	R-SCG → R-ITP	−0.1	0.1
L-LHb → L-SCG	−0.002	0.1	R-mPFC → R-NAc	0.1	0.07
			R-LHb → R-NAc	−0.08	0.1
			R-LHb → R-LHb	−0.34	−0.25
			R-ITP → R-VCa	−0.2	0.0009
			R-ITP → R-SCG	−0.09	0.2
			R-ITP → R-mPFC	0.06	0.2
			R-ITP → R-LHb	−0.0004	0.07

In the right hemisphere of male groups, EC links in both the male TRD and HC groups were intrinsic excitatory from VCa to ITP, SCG to mPFC, mPFC to NAc and ITP to mPFC (Table 4). On the contrary, EC links from VCa to LHb; NAc to mPFC and SCG; SCG to ITP; LHb to NAc; and ITP to VCa, SCG and LHb were excitatory intrinsic in one and inhibitory intrinsic in the other group. Figure 5 shows ECs with significant differences in the left hemisphere between the two male groups. Compared to the male HC group, several major EC links were identified in the male TRD group: (i) VCa excited both LHb and SCG; (ii) NAC also excited SCG, which in turn excited mPFC; (iii) NAc and mPFC excited each other; (iv) ITP inhibited VCa and LHb, which inhibited NAc; and (v) SCG and ITP inhibited each other.

## Discussion

We aimed to investigate the EC of the target brain regions for DBS in the patients with TRD, which have an important role in depression. We used the spectral DCM method to demonstrate the difference in EC of DBS target brain regions between TRD patients and healthy participants. Our results showed many major alterations in the TRD group as compared to the HC group: (i) NAc is inhibited in both hemispheres, whereas SCG and LHb are excited; (ii) mPFC is excited by SCG and ITP in the right hemisphere; and (iii) the left hemisphere in females and right hemisphere in males have a greater number of abnormal EC and a dominant role in EC of the TRD group. Furthermore, no significant association was found between EC and duration and severity of treatment resistance. This suggests that the observed EC abnormalities may not be directly influenced by these factors in patients with TRD. Our findings demonstrate that these effective connections are sex dependent and hemispherically lateralized. These findings may help identify the mechanisms underlying TRD and determine the appropriate DBS target in each patient to treat TRD.

Due to the impact of depression on brain FC, the resting state of TRD subjects is not exactly resting, being different from that of HC subjects.^53–55^ Accordingly, we extracted many links based on the EC analysis from the resting-state DCM data. Furthermore, depression depends on a variety of factors, many of which are unknown, and those that are known are accompanied by uncertainty, and their suggested underlying mechanisms are mostly controversial. Therefore, in this study, our goal was to shed light on some of these mechanisms by characterizing the EC between the major brain areas contributing to these mechanisms. Based on our findings, many mechanisms can be suggested; however, we concentrated and provided quantitative evidence for the ones consistent with the existing literature, which are discussed below.

### Abnormal EC of the target brain regions for DBS in individuals with TRD

Our results showed that compared to HC subjects, in the left hemisphere of patients with TRD, NAc excites VCa but is inhibited by ITP. NAc also excites SCG, which in turn excites LHb. In the right hemisphere, LHb is excited by VCa but inhibits both NAc and VCa, both of which inhibit ITP, which in turn inhibits SCG. However, this inhibition is weaker compared to the excitation that SCG receives from VCa. These findings highlight SCG and LHb excitation and NAc inhibition in TRD patients, consistent with MDD studies using neuroimaging approaches including PET that reported hyperactivity of SCG^56^ and LHb^57,58^ and hypoactivity of NAc.^46^

### NAc–LHb EC in individuals with TRD

Two major findings of this study were NAc inhibition and LHb excitation observed in both hemispheres of patients with TRD. NAc is located in brain areas associated with emotional, cognitive and motor control systems and functionally involved in reward processes, anhedonia and loss of motivation. Thus, NAc hypoactivity is expected to lead to multiple neurotic problems, such as depression and obsessive–compulsive disorder.^59,60^

Based on earlier studies, LHb is a key brain region in the pathophysiology of depression, and its hyperactivity,^58,61^ as observed in TRD patients in the current study, is associated with depressive symptoms, such as helplessness, anhedonia and excessive focus on the negative.^57,58,62^ When a reward is deemed inadequate and unsatisfactory by TRD patients, the firing rate of the LHb may increase, leading to dopamine release inhibition from midbrain dopaminergic neurons that project onto NAc and inhibit its activity.^62,63^

### NAc–SCG EC in individuals with TRD

Another major finding of this study was SCG hyperactivity in TRD patients. The SCG has a contributing role in the regulation of sadness and negative emotions and is metabolically overactive in patients with TRD.^56^ Previously, analyses of PET data in these patients showed that NAc–DBS increased NAc metabolism and decreased SCG metabolism.^46^ Interestingly, similar results were obtained by SCG–DBS, although NAc–DBS modulates SCG activity and SCG–DBS modulates NAc activity.^7,8^ Thus, stimulation of the SCG–NAc network by either SCG–DBS or NAc–DBS is expected to reduce depression as reported by earlier studies.^4,60,64^

### mPFC EC in individuals with TRD

Our results revealed hyperactivity of mPFC in the right hemisphere of patients with TRD due to the excitation that it receives from SCG and ITP. This is consistent with neuroimaging findings reporting the association of abnormal function of mPFC with depression^65^ and supports the strong connectivity of the SCG to mPFC in DBS therapy responders and a weak connectivity in non-responders.^15^ The mPFC plays a key role in emotional processing^66,67^ and, as expected, its hyperactivity suppressed following the SCG–DBS treatment of TRD patients.^68^

### Abnormal self-connections in individuals with TRD

Each brain region in DCM is equipped with an inhibitory self-connection. These parameters control the self-inhibition, gain and sensitivity to inputs in each region. Biologically, they can be interpreted as controlling the excitatory–inhibitory balance of the region, mediated by the interaction of pyramidal cells and inhibitory interneurons.^69,70^ Compared with the HC group, in the TRD group, a decrease in self-inhibition of the SCG and mPFC in the left hemisphere was observed, whereas in the right hemisphere, a decrease in self-inhibition of the ITP and NAc and an increase in self-inhibition were evident in the mPFC region.

### Sex dependence and hemispheric lateralization of EC in individuals with TRD

We identified the abnormal EC of specific brain regions in each hemisphere of the TRD subjects, conducting statistical analyses between the TRD and HC groups. Our results revealed substantial differences between the structure of the EC links in the two hemispheres identified by the EC analyses, indicating the strong dependence of these links on laterality.

These results demonstrated that EC alters between DBS targets from (i) females to males and (ii) the left to right hemisphere. Several important points emerged:

- In females, the left hemisphere has a greater number of abnormal EC than the right hemisphere (Fig. 4). In contrast, the right hemisphere in males reveals more abnormal effective connections (Fig. 5).
- Overall, EC abnormalities were higher in the left hemisphere of females and the right hemisphere of males.
- In females, the left hemisphere has a dominant role in the EC of the TRD group (Fig. 3), whereas the right hemisphere in males is more influential in the EC of the TRD group (Fig. 3).
- The left hemisphere has been found to be more affected by depression^71^; this may explain the higher prevalence of depression in females.

Several theories on hemispheric asymmetry for emotional processing have been proposed, including the right hemisphere hypothesis, valence hypothesis and motivational model.^72^ Based on the valence hypothesis, the left and right hemispheres are superior at processing positive and negative emotions, respectively.^73,74^ Accordingly, our results may suggest that impairments are pervasive in the recognition of positive emotions in TRD females and negative emotions in TRD males.

Our findings demonstrate that the EC between the DBS regions in TRD patients is hemispherically lateralized, supporting the findings of previous studies that indicated the association of depression with inter-hemispheric imbalance.^73–76^ Despite this, bilateral DBS remains a valuable treatment option due to its ability to address the complex interplay of brain networks, compensate for asymmetries and account for individual variability.

To the best of our knowledge, there are no comparable studies to directly compare our EC findings. However, there is existing literature suggesting potential differential gender effects of DBS for TRD. For example, Bergfeld *et al*.^77^ found that female TRD patients experienced greater improvements in depressive symptoms and quality of life compared to male TRD patients after DBS. However, this study was limited by a small sample size. Furthermore, Merkl *et al.*^78^ examined the effects of DBS in a group of patients with chronic TRD and reported that female patients had a higher response rate to DBS compared to male patients. They also found that women showed greater improvements in anxiety symptoms after DBS.

It is important to note that the evidence is limited, and more research is needed to fully understand the nature and extent of these differences. Factors such as hormonal influences, differences in brain circuitry and social factors may contribute to the observed gender effects. Further studies with larger sample sizes are necessary to confirm and better understand these gender differences in DBS outcomes for TRD.

## Conclusion

Our results identified abnormal EC between DBS targets in patients with TRD using the spectral DCM technique for the first time. The results show that both excitatory and inhibitory connections between these targets can be impaired in this patient population. The findings demonstrate that SCG and LHb are hyperactive and NAc is hypoactive. However, the nature of the excitatory/inhibitory connections and their related underlying mechanisms vary between the two hemispheres and males and females. This implies that individualizing the DBS target is required for effective DBS therapy in TRD patients, and the identification of EC can substantially contribute to this.

The clinical relevance of inter-hemispheric EC imbalance in TRD patients lies in its potential to aid in the identification of biological markers. These markers could enhance the accuracy of TRD diagnosis or help distinguish it from other mental health. Furthermore, this knowledge could contribute to the creation of more precise treatments. For example, identifying a particular imbalance could enable therapies like transcranial magnetic stimulation or DBS to be used with potential effectiveness in restoring balance. Finally, it may also provide insights into the underlying pathophysiology of TRD, helping researchers and clinicians understand why some individuals do not respond to typical treatments and what alternative treatment strategies might be effective. However, while these potential benefits are promising, it is important to note that this is a complex and evolving area of research, and more studies are needed to fully understand the implications of inter-hemispheric imbalance in TRD.

### Limitations

Major limitations of this study have been discussed. First, we did not explicitly model EC between the left and right hemispheres, that is, estimating a DCM with twelve instead of six ROIs with separate time series for each hemisphere. The reason for this was the lack of existing research regarding EC between the two hemispheres, which left us with no specific hypotheses. Second, although our study had statistically significant results, the sample size was relatively small, particularly of males. This arose from the fact that it was impractical to balance sex in the MDD group because of its prevalence among females [female-to-male ratio (2:1)].^68,79^ Despite this, sex imbalance in the MDD group was expected to have a minimal impact on the reported results. However, studies with a larger sample size are required to confirm the findings of this study, specifically about sex dependence. Third, all subjects were asked to keep their eyes closed during the resting-state fMRI scanning process. Previous studies showed slightly greater reliability when subjects were asked to rest with their eyes open.^80,81^ This may be partly due to the drowsiness of participants when allowed to rest with their eyes closed, since drowsiness has been shown to reduce reliability.^82,83^ To overcome this limitation, emphasis was placed on awake participants, and confirmation was secured after scanning was performed, indicating that they had remained awake throughout.

### Future research

The aim of this study was to evaluate the EC between TRD and normal groups. To better understand abnormal changes and their mechanisms in TRD, it is important to examine the differences between the two hemispheres. Given that this was the secondary goal of our study, we examined the changes in each hemisphere separately (due to the fact that DBS electrode implantation is bilateral), which qualitatively revealed the hemispheric differences from our results, and to examine this issue more closely, quantitative analyses are suggested to examine the differences between the two hemispheres in future studies.

We aimed to exclusively investigate the EC between DBS targets in patients with TRD. However, future studies could expand upon these findings by exploring the impact of DBS on key brain regions involved in the pathophysiology of TRD. By examining the relationship between these brain regions and the DBS targets, a more comprehensive understanding of the mechanisms underlying TRD and the therapeutic effects of DBS can be achieved. This would contribute to the advancement of knowledge in the field and enhance our ability to optimize DBS therapy for TRD patients.

Recent studies show that diffusion tensor imaging and probabilistic tractography have provided higher resolution depictions of structural connectivity between ROIs. The application of these imaging modalities to DBS neuroimaging may help address key questions about the aetiology and pathophysiology of depression and increase our understanding of the mechanisms of action of DBS from a single structure to a network level, allowing for new DBS targets to be identified and individualized DBS targeting of TRD. For example, studies based on diffusion tensor imaging have revealed that depression severity and reduced hedonic tone are associated with reduced tract volume; the former is also associated with fewer tracts in the left superolateral branch of the medial forebrain bundle.^84^ The superolateral branch of the medial forebrain bundle is a target region for DBS that is involved in both reward anticipation and perception in vertebrates.^85^ Therefore, the results of our study along with tractography and structural imaging may assist to determine the appropriate region for DBS therapy in each TRD patient, leading to more accurate and effective clinical applications, which could be the subject of future studies.

## Data Availability

Data can be made available upon reasonable request.
